# L-histidinol improves the selectivity and efficacy of alkylating agents and daunomycin in mice with P388 leukaemia.

**DOI:** 10.1038/bjc.1989.333

**Published:** 1989-11

**Authors:** R. C. Warrington, W. D. Fang

**Affiliations:** Department of Biochemistry, University of Saskatchewan, Saskatoon, Canada.

## Abstract

DBA/2J mice bearing a clonal isolate of the transplantable murine lymphocytic leukaemia line P388 were used to examine the effects of L-histidinol on the antitumour activity of three alkalyating agents (bis-chloroethylnitrosourea (BCNU), cis-diamminedichloroplatinum (II) (cisDDP) and cyclophosphamide) and the antitumour antibiotic daunomycin. Single, combined treatments with L-histidinol and either BCNU or cisDDP, at doses of the alkylating agents which were ineffective when used alone, were completely curative. Dose-response studies showed that L-histidinol conferred dose-dependent, synergistic improvements on the capacities of both BCNU and cisDDP to increase the life-span of DBA/2J mice bearing P388 leukemia. For combinations of L-histidinol and cyclophosphamide or daunomycin, two successive treatments with L-histidinol and drug were required to obtain a significant portion of long-term survivors. Thus, in this model system, the L-histidinol/anticancer drug combination approach for improving experimental cancer chemotherapy can be employed successfully with three alkylating agents and the antitumour antibiotic daunomycin.


Br. J. Cancer (1989), 60, 652–656 © The Macmillan Press Ltd., 1989

### L-Histidinol improves the selectivity and efficacy of alkylating agents and daunomycin in mice with P388 leukaemia

R.C. Warrington & W.D. Fang

*Department of Biochemistry, University of Saskatchewan, Saskatoon, Saskatchewan, Canada S7N 0W0.*

**Summary** DBA/2J mice bearing a clonal isolate of the transplantable murine lymphocytic leukaemia line P388 were used to examine the effects of L-histidinol on the antitumour activity of three alkalyating agents (*bis*-chloroethylnitrosourea (BCNU), *cis*-diamminedichloroplatinum (II) (cisDDP) and cyclophosphamide) and the antitumour antibiotic daunomycin. Single, combined treatments with L-histidinol and either BCNU or cisDDP, at doses of the alkylating agents which were ineffective when used alone, were completely curative. Dose–response studies showed that L-histidinol conferred dose-dependent, synergistic improvements on the capacities of both BCNU and cisDDP to increase the life-span of DBA/2J mice bearing P388 leukemia. For combinations of L-histidinol and cyclophosphamide or daunomycin, two successive treatments with L-histidinol and drug were required to obtain a significant portion of long-term survivors. Thus, in this model system, the L-histidinol/anticancer drug combination approach for improving experimental cancer chemotherapy can be employed successfully with three alkylating agents and the antitumour antibiotic daunomycin.

It has been argued that the adverse side-effects of anticancer drugs constitute the single most important factor in preventing their use at doses capable of curing human malignancies (Schilsky & Yarbo, 1984). Thus, a strategy which alters the action of cancer drugs such that their life-threatening toxicity for normal tissues is reduced, while their capacity to eradicate *in situ* tumour cells is enhanced, should improve the efficacy of currently available antineoplastic agents. Previous investigations from this laboratory have demonstrated that L-histidinol, a structural analogue of the essential amino acid L-histidine, can modulate cancer drug toxicity in this manner. In DBA/2J mice bearing either L1210 leukaemia (Warrington *et al.*, 1984; Warrington & Fang, 1985; Warrington, 1986) or P815 mastocytoma (Warrington *et al.*, 1987), L-histidinol eliminated the bone marrow toxicity otherwise associated with the *in vivo* use of the agents cytosine arabinoside (araC) and 5-fluorouraicil (FUra). L-Histidinol also provided a statistically significant increase in the killing of clonogenic tumour cells, in the same animals, by these two antimetabolites. These experiments also showed that L-histidinol protected tumour-free animals from supralethal doses of FUra and that the increase in selectivity of the L-histidinol/FUra combination provided parallel increases in the survival of animals bearing L1210 leukaemia (Warrington *et al.*, 1984).

Recently, other workers have begun to investigate the L-histidinol/anticancer drug combination approach for improving experimental chemotherapy. For example, the studies of Edelstein and Heilbrun (1988) with L1210 leukaemia have confirmed and, in some areas, extended our earlier findings. Ridgway and Stewart (1988) demonstrated that L-histidinol offers marked improvement in the therapeutic efficacy of some conventional antitumour agents against an advanced, solid tumour form of B16 melanoma in BDF1 mice. In contrast, a different response to FUra/L-histidinol combinations was reported by Stolfi *et al.* (1987). Although they showed that L-histidinol protected CD8F1 mice from FUra-mediated leukopenia, body weight loss and mortality, they went on to show that L-histidinol actually interfered with the capacity of FUra to eradicate the solid CD8F1 breast tumour in CD8F1 mice. Therefore, in this model, L-histidinol/FUra combinations did not confer therapeutic advantage. More recently, the same group showed that L-histidinol reduced FUra incorporation into RNA in the normal mouse tissue and in the CD8F1 breast tumour (Sawyer *et al.*, 1988). The authors suggested that L-histidinol is an effective modulator of FUra toxicity in those types of tumours wherein the major mode of action of FUra is to inhibit thymidylate synthetase via its metabolic conversion to 5-fluorodeoxyuridine. The latter mode is presumed to be the case for the tumour models studied in this laboratory.

The objective of this study was to determine whether L-histidinol would modulate the action of other classes of antineoplastic agents in the same manner as has been shown for antimetabolites. Of particular interest were the alkylating agents and the anthracycline antibiotics, which share a number of attributes, the most important of which is remarkable clinical effectiveness (Chabner & Meyers, 1983). If L-histidinol could be shown to modulate the responses of normal and tumorous tissues to these agents, so as to protect the former tissues while increasing the vulnerability of the latter, then the already demonstrated efficacy of the alkylating agents and anthracyclines might be improved further. Accordingly, the effects of L-histidinol on the activity of three alkylating agents and an anthracycline in the treatment of P388 leukaemia in DBA/2J mice were investigated.

### Materials and methods

### *Animals, injection protocols and media*

Female DBA/2J mice, of 20–25 g body weight, were used for all experiments. L-Histidinol dihydrochloride (Sigma Chemical Co., St Louis, MO) was dissolved in water, and the pH of the resultant solution was adjusted to 7.3 and brought to a final concentration of 100 mg ml$^{-1}$. Stock solutions were filter-sterilised, and stored frozen until needed. All injections were administered intraperitoneally (i.p.) to non-anaesthetised mice. Injection schedules and doses for specific experiments are itemised in the figure legends. Media, sera and Dulbecco's phosphate-buffered saline were products of Grand Island Biological Co. Canada Inc. (Burlington, Ontario).

### *P388 leukaemia cells,* in vitro *propagation and clonogenic assays of intrafemoral P388 leukaemia and CFU-C/GM*

The P388 lymphocytic leukaemia/MR1 line was provided generously by EG and G Mason Research Institute. A clonal isolate was picked from soft agar, propagated in bulk and frozen down as a stock which was used for all experiments described herein. The isolate (hereafter simply referred to as P388 leukaemia) was maintained in RPMI 1640 medium supplemented with 10% (v/v) fetal bovine serum. *In vivo* assessments of responses of CFU-C/GM and intrafemoral P388 cells employed the previously described intrafemoral tumour model (Warrington & Fang, 1985; Warrington, 1986). For the saline/BCNU and L-histidinol/BCNU-treated groups, each determination of intrafemoral tumour and CFU-C/GM

Correspondence: R.C. Warrington.
Received 25 January 1989; and in revised form 24 April 1989.

responses required the femurs of six mice. For the L-histidinol/drug-treated and saline-treated groups, the femurs of four and two mice, respectively, were employed.

*Animal survival experiments and drug treatment protocols*

Appropriate numbers of DBA/2J mice were injected intraperitoneally with 1 × 10$^6$ P388 leukaemia cells. For evaluations using the *early treatment mode,* the animals were divided randomly into treatment groups (six per group) 24 h later, and subsequently treated, as described below. For evaluations using the *delayed treatment mode,* the protocol was the same, except that grouping and drug treatments were instigated 72 h after the tumour cells had been injected. A standard drug treatment protocol was used in all cases, and is as follows: 24 (or 72) hours after i.p. injection of tumour cells, the animals were placed, randomly, into treatment groups of six which received saline; drug; both L-histidinol and drug (drug was given at 0 time; L-histidinol was given in five consecutive 5 mg injections at −2, 0, 2, 4, and 6 hours) or L-histidinol alone (given five times in 5 mg injections, as above). The concentrations of anticancer drug employed is shown in each legend; the L-histidinol was given as described, except where noted otherwise. Thereafter, the number of surviving animals were scored at 24-h intervals. The pooled results of two independent determinations are shown in the figures. Accumulated data for various determinations are shown in Tables I and II.

**Table I** Summary of medial survival times and number of 60 day survivors for various treatments of DBA/2J mice bearing P388 leukaemia

| *Treatment (mg per animal)* | *Median survival time (days)* | *60 day survivors* |
|---|---|---|
| None | 7 ± 0.4; *n* = 16 | 0 (out of 96) |
| L-Histidinol[a] | 8.7 ± 1.1; *n* = 12 | 0 (out of 72) |
| BCNU (1 mg) | 8; 8; 8; 7 | 0; 0; 0; 0[b] |
| BCNU + L-histidinol | –; –; –; – | 6; 6; 6; 6 |
| BCNU (1 mg)[c] | 18; 17 | 0; 0 |
| BCNU + L-histidinol | –; – | 5; 5 |
| CisDDP (0.2 mg) | 7; 8 | 0; 0 |
| CisDDP + L-histidinol | –; – | 5[d];6 |
| CisDDP (0.2 mg)[c] | 16; 15 | 0; 0 |
| CisDDP + L-histidinol | –; – | 4; 5 |
| Daunomycin (0.02 mg) | 8; 7 | 0; 0 |
| Daunomycin + L-histidinol | 14; 13 | 0; 0 |
| Daunomycin (0.02 mg)[e] | 8; 11 | 0; 0 |
| Daunomycin + L-histidinol | –; – | 5; 5 |
| Cyclophosphamide (3 mg) | 11; 13 | 0; 0 |
| Cyclophosphamide + L-histidinol | 19; 31 | 2; 2 |
| Cyclophosphamide (3 mg)[e] | 12; 15 | 0; 0 |
| Cyclophosphamide + L-histidinol | –; – | 5; 5 |

Unless stated otherwise, groups of 6 animals were given 1 × 10$^6$ cells i.p.; 24 h later treatments were instigated (see Materials and methods). [a]Standard protocol (5 × 5 mg; see Materials and methods). [b]Number out of 6 animals per group. [c]Late treatment. [d]One animal died on day 55. [e]Two injections, given on day 1 and day 8.

**Table II** Medial survival times and number of 60 day survivors for BCNU and L-histidinol/BCNU treatment of mice bearing other tumours

| *Treatment (mg per animal)* | *Median survival time (days)* | *60 day survivors*[b] |
|---|---|---|
| *DBA/2J mice with L1210 leukaemia*[a] | | |
| None | 8; 7 | 0; 0 |
| L-Histidinol | 9; 9 | 0; 0 |
| BCNU (1 mg) | 9; 7 | 0; 0 |
| BCNU + L-histidinol | 32; 28 | 2; 3 |
| *C57/BL mice with B16f10 melanoma*[c] | | |
| None | 16; 18; 17 | 0; 0; 0 |
| L-Histidinol | 18; 29; 21 | 0; 1; 1 |
| BCNU (1 mg) | 30; 29; 28 | 1; 2; 0 |
| BCNU + L-histidinol | –; –; – | 6; 6; 6 |

[a]1 × 10$^6$ cells injected intraperitoneally; treatments (as described in Table I) instigated 24 h later. [b]Number out of 6 animals per group. [c]5 × 10$^5$ cells injected intravenously; treatments (as described in Table I) instigated 24 h later.

### Results

*Increased selectivity of BCNU provided by histidinol/BCNU combination in leukaemic mice*

The intrafemoral tumour model developed in this laboratory (Warrington & Fang, 1985; Warrington, 1986) was employed to evaluate the effect of L-histidinol on the toxicity of BCNU, cyclophosphamide, daunomycin and adriamycin. Figure 1 shows the results of such an evaluation for BCNU. Figure 1a displays the responses of the normal marrow cell population; Figure 1b panel shows the responses of the intrafemoral P388 leukaemia cells. The toxicity of BCNU for the normal marrow cells in the leukaemic marrows is virtually eliminated by the inclusion of L-histidinol (Figure 1a). The survival of CFU-C/GM in the L-histidinol/BCNU group was significantly different from that of the saline/BCNU

**Figure 1** Differential responses of murine bone marrow cells (*a*) and intrafemoral P388 leukaemia cells (*b*) to L-histidinol and BCNU combinations. Appropriate numbers of tumour-bearing mice received saline (O); BCNU (1 mg per mouse; ▲); BCNU and L-histidinol (1 mg per ml mouse of BCNU at 0 time and five consecutive 5 mg injections of L-histidinol at −2, 0, 2, 4, and 6 h; Δ) or L-histidinol alone (Δ; five times 5 mg injections, as described in Materials and methods). At 24, 48, 72 h post-treatment, clonogenic normal marrow cells (CFU-C/GM; *a*) or intrafemoral clonogenic P388 leukaemia cells (*b*) were scored. The values shown are means ± s.e.m. for three determinations.

group (at 24, 48 and 72 h, $P<0.001$; Student's $t$ test). Figure 1b demonstrates that L-histidinol accentuated, simultaneously, the susceptibility of intrafemoral P388 leukaemia cells to the drug BCNU. The increased eradication of clonogenic tumour cells mediated by the L-histidinol/BCNU combination was significant compared to that observed in the saline/BCNU group ($P<0.001$ at the three assay times). These results demonstrate that L-histidinol conferred a significant increase in the selectivity of BCNU in mice bearing intrafemoral P388 leukaemia cells. Although not shown, qualitatively identical results were obtained for combinations of L-histidinol and cyclophosphamide, adriamycin and daunomycin; in all cases, L-histidinol eliminated the toxicity that these agents otherwise had for the CFU-C/GM and, simultaneously, increased their capacity to kill clonogenic P388 leukaemia cells resident in the femoral cavities (not shown).

*Curative treatment of DBA/2J mice bearing lethal burdens of P388 leukaemia with either BCNU or cisDDP and L-histidinol*

Experiments were performed to assess what impact, if any, the various L-histidinol/drug combinations studied above would have on the survival of animals bearing P388 leukaemia. The first agent tested was BCNU. As Figure 2 reveals, P388 leukaemia ($1 \times 10^6$ cells, injected intraperitoneally) killed all untreated animals within 8 days of injection. Mice treated 24 h after tumour challenge with either L-histidinol or BCNU (1 mg per animal) survived only marginally longer (9–15 days). Remarkably, a single L-histidinol/BCNU regimen, using 1 mg BCNU per animal, proved to be 100% curative (see Table I also). Similarly, cisDDP (0.4 mg per animal) and L-histidinol proved to be curative as well; animals treated with the same dose of cisDDP without L-histidinol co-treatment were all dead within 16 days (Table I).

*Improved treatment in a delayed model using L-histidinol and either BCNU or cisDDP*

In order to assess the efficacy of BCNU/histidinol and cisDDP/histidinol combinations under more rigorous conditions, a delay of 72 h in the onset of treatment was used. Even though this treatment mode provides a larger tumour burden at the onset of treatment, L-histidinol was able to improve the efficacy of BCNU and cisDDP (Table I) to the extent that 10 of 12 and 9 of 12 tumour-bearing animals, respectively, became long-term survivors. Although it is not clear why, both L-histidinol and BCNU, on their own, provided better increases in survival in this delayed mode than they did with the early mode.

*Dose–response studies of L-histidinol/BCNU and L-histidinol/cisDDP combinations*

Given the remarkable capacity of L-histidinol to improve the efficacy of both BCNU and cisDDP demonstrated above, it was of interest to study the interaction between these agents. Various doses of L-histidinol and the two drugs, both alone and in varying combinations, were given to P388 leukaemia-bearing animals 24 h after tumour cell inoculation. After evaluating the median day of death for each group, per cent increases in median survival time were calculated and plotted (Figures 3 and 4). Because optimum treatments were 100% curative, arbitrary end-points of 600% and 1000% increases in median survival time were used for the BCNU and the cisDDP experiments respectively. (The basis for choosing these particular end-points was for graphical convenience only.) Figures 3 and 4 both show that, on their own, the three doses of L-histidinol tested (five 2-hourly injections of 1.25, 2.5 or 5 mg per animal) had a minor effect on survival. Figure 3 shows that L-histidinol has a modest, probably additive effect on the lower doses of BCNU tested. However, as the dose of BCNU employed was increased, the relative effect of increasing doses of L-histidinol also increased.

At the highest dose of BCNU tested (1 mg per animal), the three concentrations of L-histidinol tested had an increasing, eventually synergistic, effect of survival time. Similar effects are evident in Figure 4, which shows the results of an analysis of the interactions between L-histidinol and cisDDP. In this case, however, it is clear that the drug and L-histidinol are interacting in a more complex manner than was observed for BCNU and L-histidinol. For the two lower doses of cisDDP used, L-histidinol has a dose-dependent and synergistic effect on survival times. For the highest dose of L-histidinol with the two lower cisDDP doses, the interaction was synergistic and totally curative. The highest dose of cisDDP employed did not give improved survival, presumably because of toxicity; this toxicity was partially countered only by the highest dose of L-histidinol.

**Figure 2** Effect of L-histidinol/BCNU combinations on the survival of DBA/2J mice bearing P388 leukaemia. Treatment groups (of six) received saline (O); BCNU (Δ; 1 mg per animal); L-histidinol/BCNU (□; five 5 mg injections and 1 mg injection, respectively); or L-histidinol (□; five 5 mg injections). Thereafter, the number of surviving animals were scored at 24 h intervals. The pooled results of two independent determinations are shown.

**Figure 3** Dose–response determination for L-histidinol and BCNU combinations. Animals were treated as in the legend to Figure 2. Groups of animals were given five treatments with 1.25 (O), 2.5 (Δ) or 5 (□) mg per animal of L-histidinol, without or with the indicated doses of BCNU. The responses observed with BCNU alone are indicated with the solid symbols. The per cent increase in median survival times for the various drug combinations are plotted.

**Figure 4** Dose–response determination for L-histidinol and cisDDP combinations. Animals were treated as in the legend to Figure 3, except that cisDDP (at the indicated levels) replaced BCNU. The per cent increase in median survival times for the various drug combinations are plotted.

*Improved treatment with two courses of L-histidinol and either cyclophosphamide or daunomycin*

Combinations of L-histidinol and either cyclophosphamide or daunomycin were also assessed by survival experiments. Although these agents were less influenced by L-histidinol than were BCNU and cisDDP, the combinations proved nevertheless to be efficacious. As is seen in Figure 5 and Table I, no long-term survivors were observed for single or double treatments with either of these drugs on their own. With both drugs, single histidinol/drug regimens gave improved 50% survival time compared to the single drug-alone treatments and, in the case of cyclophosphamide, the single combination regimen gave four of 12 long-term survivors (Figure 5 and Table I). Two consecutive courses of L-histidinol and either cyclophosphamide or daunomycin cured 10 of 12 tumour-bearing animals. Thus, by combining L-histidinol and cyclophosphamide or daunomycin at levels which were ineffective when used alone, more than 80% of the mice bearing P388 leukaemia were cured with two consecutive treatments.

*Improved treatment of other tumour models with L-histidinol and BCNU*

It was of interest to determine whether the response of the P388 line to histidinol/BCNU treatment would be observed with other transplantable tumours. Further assessments of the efficacy of the L-histidinol/BCNU combination were carried out in DBA/2J mice bearing intraperitoneal L1210 leukaemia and C57/BL mice bearing pulmonary B16f10 melanoma (Table II). With L1210 leukaemia, L-histidinol and BCNU gave five out of 12 long-term survivors at a dose of BCNU which was completely ineffective on its own (Table II). A single course of L-histidinol and BCNU cured 100% of C57/BL mice of disseminated B16f10 melanoma (Table II) at a dose of BCNU which cured three of 18 animals on its own. Thus, the combination of L-histidinol and BCNU appears to be a particularly effective combination for treating transplantable murine tumours *in situ*, whether these are related to P388 leukaemia or not.

### Discussion

Two significant observations are made in this study. The first is that L-histidinol increases the selectivity of both alkylating agents and an antitumour antibiotic in this experimental model. Figure 1 demonostrates this capacity for BCNU; the analogue eliminates the toxicity BCNU otherwise possess for the CFU-C/GM and increases the ability of the drug to

**Figure 5** Effect of L-histidinol/cyclophosphamide combinations on the survival of DBA/2J mice bearing P388 leukaemia. Treatment groups received saline (O), cyclosphosphamide (3 mg per animal on day 1 only (●), or on days 1 and 8 (▲)) or L-histidinol and cyclophosphamide (five 5 mg injections and 3 mg, respectively, either on day 1(□) or on both day 1 and day 8(∇). Thereafter the number of surviving animals were scored at daily intervals. The pooled results of two independent determinations are shown.

eradicate P388 leukaemia cells resident in the same tissue. Similar modulations of the toxicity of cyclophosphamide, adriamycin and daunomycin mediated by co-administration of L-histidinol were observed with the intrafemoral model. The second observation, which is presumed to be a consequence of this improved selectivity, is that L-histidinol also improves the efficacy of the three alkylating agents and the antitumour antibiotic tested in the P388 leukaemia/DBA/2J mouse model. The improvement is most dramatic with BCNU and cisDDP. Combining either of these alkylating agents and L-histidinol, at levels which were completely ineffective when used alone, proved to be 100% curative for P388 leukaemia (Figures 2, 3 and 4). Dose–response studies (Figures 3 and 4) showed that L-histidinol conferred dose-dependent, synergistic improvements on the capacities of both BCNU and cisDDP to increase the life-span of DBA/2J mice bearing P388 leukaemia. Although the response was neither as dramatic nor as complete as with BCNU and cisDDP, L-histidinol nevertheless improved significantly the efficacy of another alkylating agent, cyclophosphamide, and the anthracycline daunomycin in this experimental tumour model. With these agents, two courses in combination with L-histidinol were required to obtain >50% long-term survivors (Figure 5 and Table I). As was the case with BCNU and cisDDP, the doses of these two drugs which proved to be curative for >50% of the animals when used in conjunction with L-histidinol gave no long-term survivors when employed alone.

Because the studies reported herein employed a clonal isolate of P388, the 'standard' P388 leukaemia line maintained by the USNCI/DTP tumour repository was obtained. After expansion in DBA/2J mice, this form of P388 leukaemia was injected into animals and challenged with BCNU and L-histidinol/BCNU combinations. This non-cloned form of P388 was less aggressive, but much more responsive to BCNU, than was its cloned counterpart, since the 50% survival time of DBA/2J mice bearing the standard P388 leukaemia was extended from 12 to 40 days with a single treatment with BCNU (1 mg per animal). However, only one out of six animals in this group became a long-term survivor. In contrast, all animals bearing the standard, animal-passaged P388 leukaemia which were treated with L-histidinol and BCNU survived (data not shown). Thus, the marked synergy between L-histidinol and BCNU observed in Figure 2 does not appear to be due to some peculiar attribute of that particular isolate. This notion is further supported by the data in Table II, which show that other transplantable murine tumours also respond well to L-histidinol and BCNU.

We have recently demonstrated that L-histidinol has the capacity to overcome the multidrug resistant phenotype in two cell lines which over-express P-glycoprotein (Warrington & Fang, 1989). This observation, coupled with the findings reported in this paper, suggest that there are means not only of improving the therapeutic indices of commonly used antineoplastic agents, but also of overcoming the problem of multidrug resistance in experimental systems. Whether L-histidinol, or some other agent, will achieve these effects in the clinical situation remains to be demonstrated.

This work was supported by a grant from the National Cancer Institute of Canada. The authors thank Mrs S. Bueckert, Ms B. Britton and Ms E. Behr for their assistance in preparing this manuscript.

### References

CHABNER, B.A. & MEYERS, C.T. (1983). Clinical pharmacology of cancer chemotherapy. In *Cancer Principles and Practice of Oncology* (2nd edn), DeVita, V.T., Hellman, S. & Rosenberg, S.A. (eds) p. 304. Lippincott: Philadelphia.

EDELSTEIN, M. & HEILBRUN, L.K. (1988). Specificity, schedule and proliferation dependence of infused L-histidinol after 5-fluorouracil in mice. *Cancer Res.*, **48**, 1470.

RIDGWAY, H.R. & STEWART, D.P. (1988). L-Histidinol improves the therapeutic efficacy of some conventional antitumor agents against the advanced B16 melonama in mice. *Proc. Am. Assoc. Cancer Res.*, **29**, abstract 1908.

SAWYER, R.C., STOLFI, R.L. & MARTIN, D.S. (1988). Effect of L-histidinol on the metabolism of 5-fluorouracil in the BALB/c × DBA/8 F1 murine model system. *Cancer Res.*, **48**, 6664.

SCHILSKY, R.L. & YARBO, T.W. (1984). Pharmacology of antineoplastic drugs. In *Toxicity of Chemotherapy*, Perry, M.C. & 'Yarbo, J.W. (eds) p.21. Grune and Stratton: New York.

STOLFI, R.L., SAWYER, R.C. & MARTIN, D.S. (1987). Failure of L-histidinol to improve the therapeutic efficiency of 5-fluorouracil against murine breast tumours. *Cancer Res.*, **47**, 16.

WARRINGTON, R.C. (1986). A novel approach for improving the efficacy of experimental cancer chemotherapy using combinations of anticancer drugs and L-histidinol. *Anticancer Res.*, **6**, 451.

WARRINGTON, R.C., CHENG, I. & FANG, W.D. (1987). Effects of L-histidinol on the susceptibility of P815 mastocytoma cells to selected anticancer drugs in vitro and in DBA/2J mice. *J. Natl Cancer Inst.*, **78**, 1177.

WARRINGTON, R.C. & FANG, W.D. (1985). Histidinol mediated enhancement of the specificity of two anticancer drugs in mice bearing leukemic bone marrow disease. *J. Natl Cancer Inst.*, **74**, 1071.

WARRINGTON, R.C. & FANG, W.D. (1989). Reversal of the multidrug resistant phenotype of Chinese hamster ovary cells by L-histidinol. *J. Natl Cancer Inst.*, **81**, 798.

WARRINGTON, R.C., MUZYKA, T.G. & FANG, W.D. (1984). Histidinol-mediated improvement in the specificity of 1-β-D-arabinofuranosylcytosine and 5-fluorouracil in L1210 leukemia-bearing mice. *Cancer Res.*, **44**, 2929.


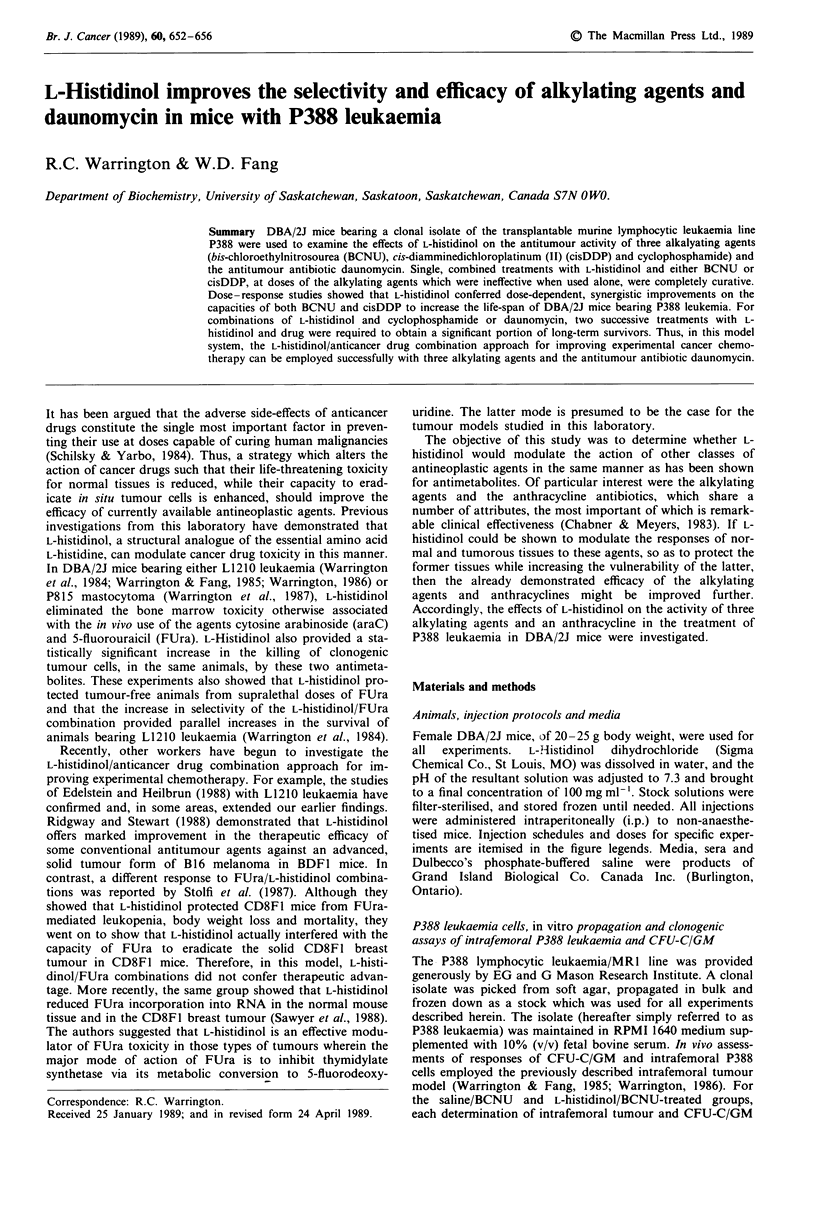

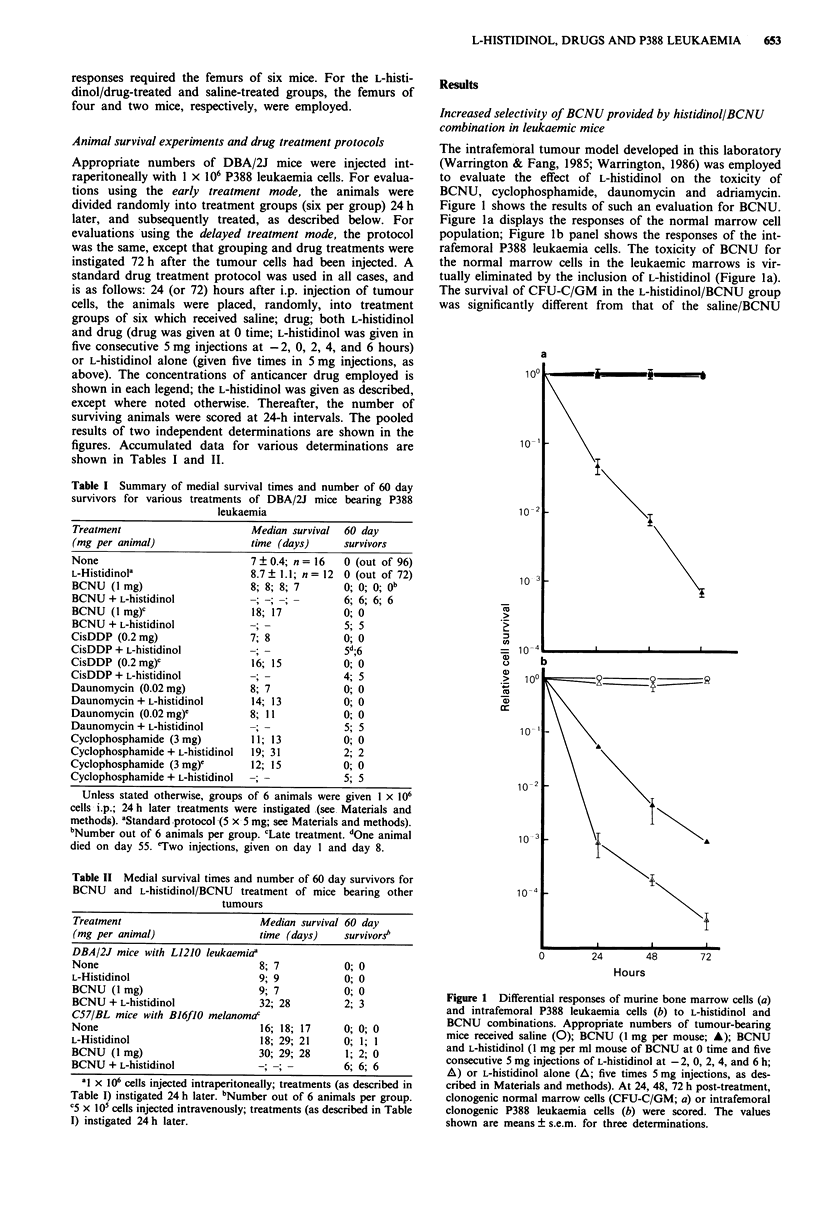

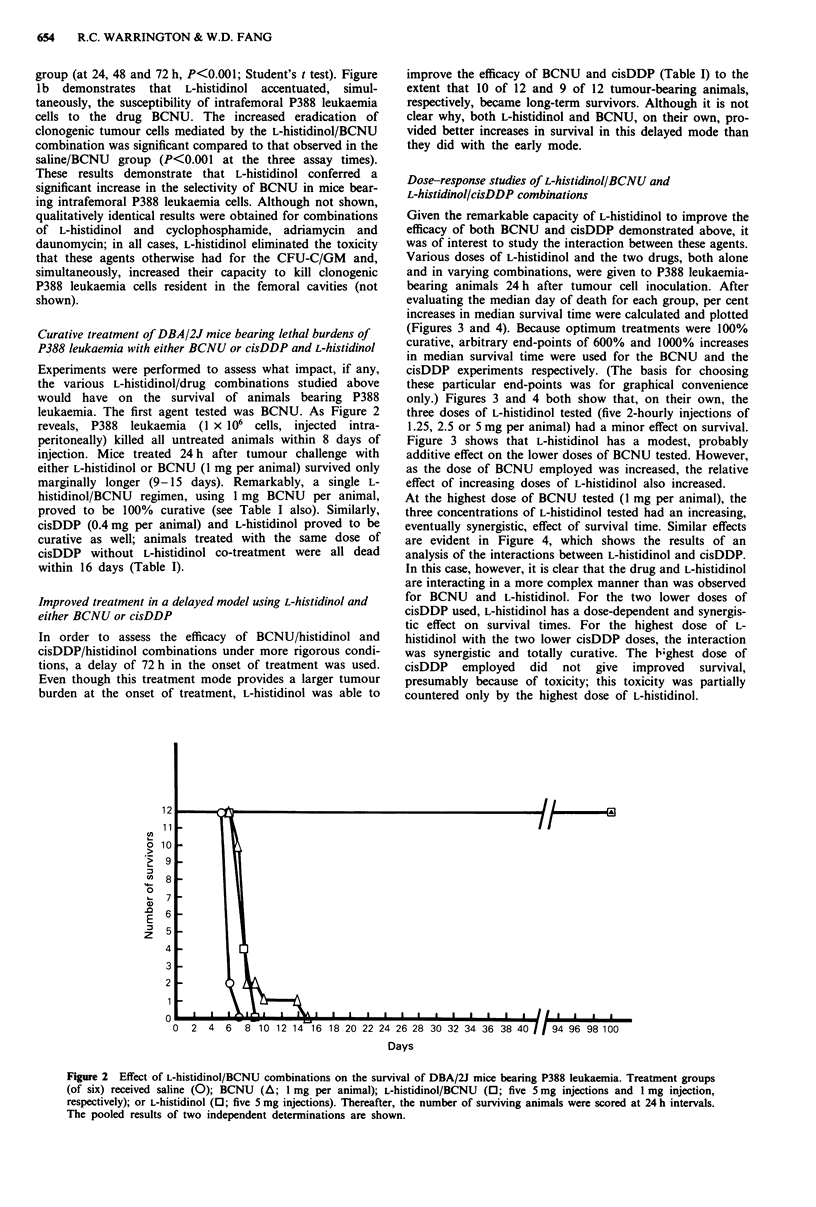

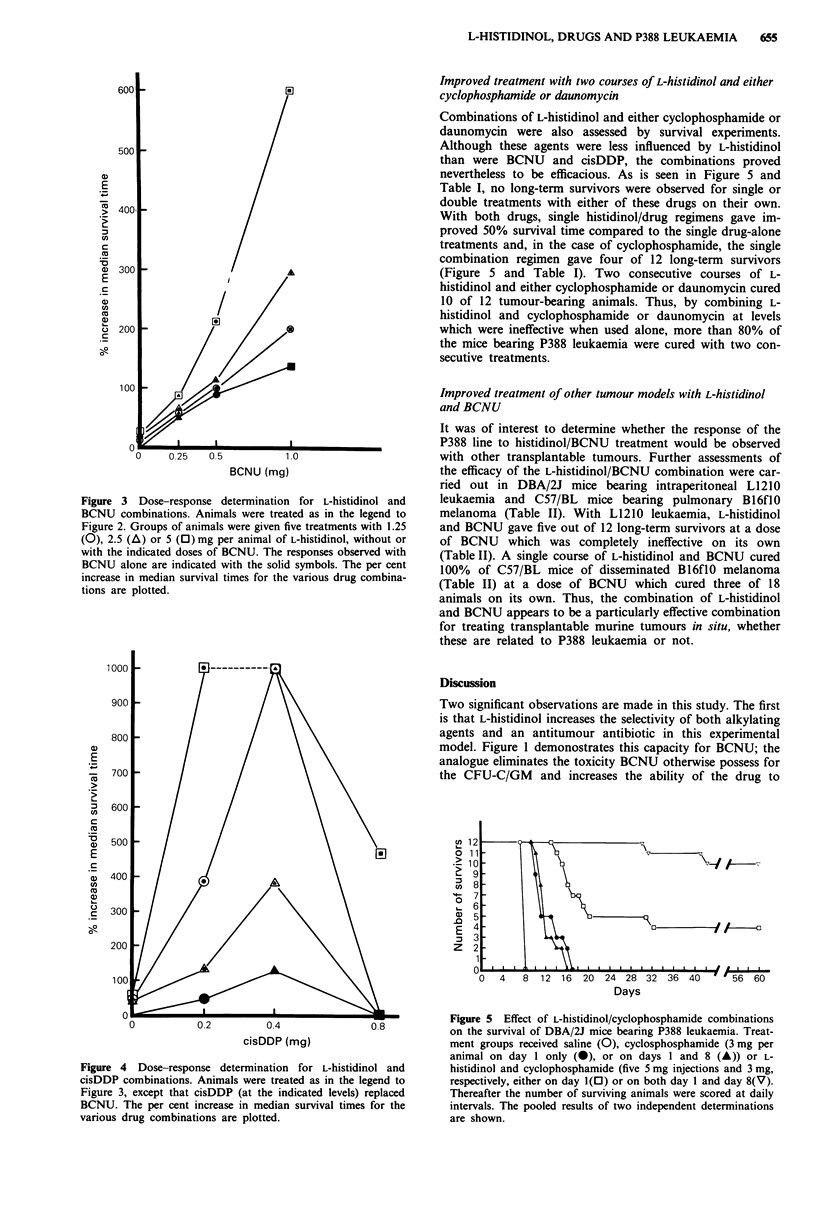

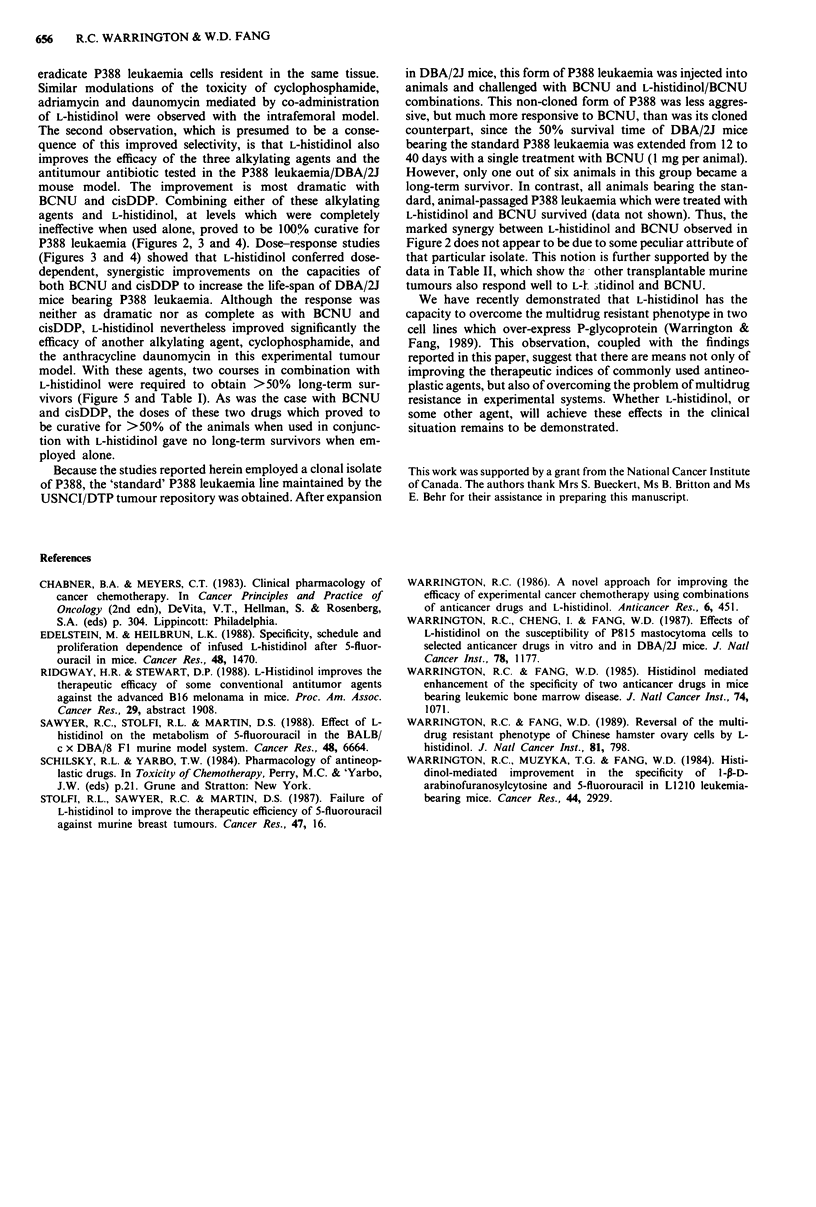


## References

[OCR_00634] Edelstein M. B., Heilbrun L. K. (1988). Specificity, schedule, and proliferation dependence of infused L-histidinol after 5-fluorouracil in mice.. Cancer Res.

[OCR_00645] Sawyer R. C., Stolfi R. L., Martin D. S. (1988). Effect of L-histidinol on the metabolism of 5-fluorouracil in the BALB/c x DBA/8 F1 murine tumor system.. Cancer Res.

[OCR_00655] Stolfi R. L., Sawyer R. C., Martin D. S. (1987). Failure of L-histidinol to improve the therapeutic efficiency of 5-fluorouracil against murine breast tumors.. Cancer Res.

[OCR_00660] Warrington R. C. (1986). A novel approach for improving the efficacy of experimental cancer chemotherapy using combinations of anticancer drugs and L-histidinol.. Anticancer Res.

[OCR_00665] Warrington R. C., Cheng I., Fang W. D. (1987). Effects of L-histidinol on the susceptibility of P815 mastocytoma cells to selected anticancer drugs in vitro and in DBA/2J mice.. J Natl Cancer Inst.

[OCR_00671] Warrington R. C., Fang W. D. (1985). Histidinol-mediated enhancement of the specificity of two anticancer drugs in mice bearing leukemic bone marrow disease.. J Natl Cancer Inst.

[OCR_00677] Warrington R. C., Fang W. D. (1989). Reversal of the multidrug-resistant phenotype of Chinese hamster ovary cells by L-histidinol.. J Natl Cancer Inst.

[OCR_00682] Warrington R. C., Muzyka T. G., Fang W. D. (1984). Histidinol-mediated improvement in the specificity of 1-beta-D-arabinofuranosylcytosine and 5-fluorouracil in L 1210 leukemia-bearing mice.. Cancer Res.

